# Clinical characteristics, treatment patterns and long-term outcomes of warm and mixed autoimmune hemolytic anemia: insights from a real-world study

**DOI:** 10.3389/fimmu.2026.1931581

**Published:** 2026-10-12

**Authors:** Junan Zhang, Yuan Li, Jiewen Qiu, Yizhou Zheng, Meili Ge, Fengkui Zhang, Jun Shi, Xin Zhao

**Affiliations:** 1 State Key Laboratory of Experimental Hematology, National Clinical Research Center for Blood Diseases, Haihe Laboratory of Cell Ecosystem, Institute of Hematology & Blood Diseases Hospital, Chinese Academy of Medical Sciences & Peking Union Medical College, Tianjin, China; 2 Tianjin Institutes of Health Science, Tianjin, China

**Keywords:** autoimmune hemolytic anemia, mixed AIHA, prognosis, real-world study, treatment patterns, warm AIHA

## Abstract

**Objective:**

Autoimmune hemolytic anemia (AIHA) represents a heterogeneous group of autoimmune disorders, with limited large-scale real-world evidence from Asian populations. This study aimed to characterize the clinical characteristics, treatment patterns, and long-term outcomes of patients with warm AIHA (wAIHA) and mixed AIHA (mAIHA).

**Methods:**

This single-center retrospective study screened 629 patients with AIHA hospitalized in China between 2014 and 2023. After excluding 32 patients with cold AIHA and 31 with DAT-negative AIHA, 566 patients (449 wAIHA and 117 mAIHA) were included, with a median follow-up of 57 months.

**Results:**

Compared with wAIHA, mAIHA patients presented with earlier onset, poorer bone marrow compensation, and cold AIHA-like features despite comparable hemoglobin levels. Secondary AIHA was more prevalent in mAIHA (39.3% vs. 22.5%). Glucocorticoids remained the first-line treatment with similar response rates in both subtypes, while rituximab was the predominant second-line therapy. Treatment strategies and outcomes with multiple emerging therapies were also summarized. Median event-free survival was 17 months, with relapses mainly occurring during treatment tapering or infections. Estimated overall survival (OS) rates at 1, 3, and 5 years were 96%, 92%, and 89%, respectively, with infections and disease progression being leading causes of mortality. Independent risk factors associated with OS included age ≥60, hemoglobin at onset <6 g/dL, bone marrow responsiveness index <121, male sex, and an IgM+IgA-mediated serological pattern, whereas cold agglutinin titers demonstrated limited value in predicting OS or treatment response.

**Conclusions:**

mAIHA and wAIHA share comparable glucocorticoid response and long-term survival despite distinct clinical and serological characteristics. This large real-world cohort delineates the profiles of wAIHA and mAIHA and identifies baseline factors associated with OS to support individualized management. Emerging therapies present promising prospects in AIHA management.

## 1 Introduction

Autoimmune hemolytic anemia (AIHA) is a group of anemias characterized by autoantibody-mediated destruction of red blood cells (RBCs). As the most prevalent form of acquired hemolytic anemia, AIHA is marked by its clinical heterogeneity, unpredictable disease progression, high relapse rates and thrombotic risk (1–4). Based on underlying etiology, AIHA is divided into primary and secondary forms (5). Direct antiglobulin test (DAT) and cold agglutinin (CA) level remain the diagnostic cornerstones for AIHA, guiding its major subclassification into: (1) warm AIHA (wAIHA, 37 °C), (2) cold AIHA (cAIHA, ≤4 °C), (3) mixed AIHA (mAIHA), and (4) DAT-negative AIHA, which reflects the optimal temperature at which antibodies bind to RBCs (6, 7). Paroxysmal cold hemoglobinuria, however, is recognized as a distinct entity diagnosed by biphasic Donath-Landsteiner antibody. Among secondary AIHAs, autoimmune disorders (AIDs) and lymphoproliferative diseases (LPDs) constitute the majority of cases, followed by solid tumors, infections, drugs, immunodeficiencies, and hematopoietic stem cell transplantation (1, 2, 8).

As the most prevalent form, wAIHA arises from immune tolerance breakdown characterized by peripheral and splenic T helper 17/regulatory T cell imbalance (9, 10). The immune dysregulation drives autoreactive B-cell expansion and subsequent polyclonal plasma cell differentiation, which generate pathogenic antibodies (primarily IgG, though IgA or warm-reactive IgM may also involve) targeting Rh antigens, band 3, and glycophorin A on RBCs (11, 12). In the spleen, opsonized RBCs bind to Fcγ receptors (FcγR) on resident macrophages, triggering antibody-dependent cellular phagocytosis and cytotoxicity (1, 13). Incomplete phagocytosis of opsonized RBCs leads to spherocytosis in approximately 40% of cases (14). Besides, IgG1/IgG3 activate the classical complement pathway, leading to membrane attack complex formation and complement-dependent cytotoxicity (15).

Glucocorticoids (GCs) remain the standard first-line therapy for wAIHA. For patients presenting with severe manifestations (abrupt onset, hemoglobin [Hb] <6 g/dL, Evans syndrome, IgA-mediated, or DAT-negative status) or for elderly patients with significant comorbidities, the early addition of RTX to GCs is frequently considered (1, 2, 16, 17). While RTX has established its role in relapsed/steroid-refractory wAIHA, the optimal sequencing of other second-line therapies, including azathioprine (AZA), mycophenolate mofetil (MMF), cyclosporine A (CsA), and cyclophosphamide (CTX) remains controversial (17). Splenectomy has been relegated to a later-line option due to risks of life-threatening infections and thrombotic events (18). Recent advances have broadened the treatment landscape for refractory wAIHA (17, 19). Ongoing prospective trials are evaluating promising therapies, including spleen tyrosine kinase (Syk) inhibitors (20, 21), Bruton tyrosine kinase (BTK) inhibitors (19, 22), plasma cell targeting therapies (bortezomib, daratumumab) (23), mammalian target of rapamycin (mTOR) inhibitor sirolimus (SRL) (24), B-cell activating factor (BAFF) inhibitors (25), phosphatidylinositol 3-kinase delta (PI3Kδ) inhibitors (26), and notably, chimeric antigen receptor (CAR)-T cell (27) along with B-cell maturation antigen (BCMA)-targeted T-cell-engager therapy (28). The management of mAIHA lacks consensus due to limited prospective studies (29, 30) and mostly follows the treatment protocol of wAIHA.

Despite existing studies, critical gaps remain in delineating AIHA’s real-world clinical heterogeneity, treatment outcomes, and long-term prognosis, particularly in Asian populations (31, 32) where real-world data from large-scale retrospective studies remain absent. Here, we performed a retrospective analysis of Chinese patients with wAIHA and mAIHA, focusing on comparative clinical, serological, therapeutic, and long-term outcomes, while also systematically examining complication patterns and prognostic factors to provide real-world evidence for individualized management.

## 2 Materials and methods

### 2.1 Study population and ethics

In this single-center retrospective study, we screened 629 AIHA patients hospitalized at the Blood Diseases Hospital, Chinese Academy of Medical Sciences and Peking Union Medical College from January 2014 to December 2023. The diagnosis required anemia with evidence of hemolysis (elevated lactate dehydrogenase [LDH], indirect bilirubin [IBIL], and free hemoglobin [FHb]; decreased haptoglobin [HAP]; peripheral blood smear findings) and positive DAT (or rigorous differential diagnosis plus response to immunosuppressive therapy for DAT-negative cases). For DAT-positive AIHA, cases were subclassified as wAIHA (CA titer <64; DAT typically positive for IgG or IgG+C3, less commonly isolated C3), cAIHA (CA titer ≥ 64; DAT typically showing isolated C3 positivity), or mAIHA. Given the unavailability of thermal amplitude assessment, mAIHA was operationally defined by the presence of IgG positivity together with CA titers ≥ 64, based on reported clinical criteria (16, 33). CA titers were determined by serial serum dilution from 1:2 to 1:2048. After incubation with washed RBCs at 4°C for 2 hours, the highest dilution showing visible agglutination was recorded as the titer. For all patients, DAT was performed via a microcolumn gel card technique. Briefly, 50 μL of a 1% patient RBC suspension was added to each well of a DC-Screening I card and centrifuged at 1030 r/min for 10 minutes. Complete RBC deposition at the column bottom indicated a negative result; otherwise, it was considered positive. This procedure entailed an initial polyspecific anti-human globulin screening followed by monospecific anti-IgG, -IgM, -IgA, and -C3 testing. IgM and IgA positivity provided additional serological information but was not solely used to determine AIHA subtype classification. Patients with cAIHA (n=32) or DAT-negative AIHA (n=31) were excluded. Finally, 566 patients were enrolled in this study. A standardized unified follow-up was completed for all patients in April 2025. This study received ethical approval from the Ethics Committee of the Institute of Hematology and Blood Diseases Hospital, Chinese Academy of Medical Sciences and Peking Union Medical College. All study procedures complied with the Declaration of Helsinki (2024 revision). Written informed consent was acquired from all participants and/or their legally authorized representatives.

### 2.2 Study definitions

The definition of Evans syndrome (ES) has recently been revised to require immune-mediated cytopenias affecting any two blood cell lineages (34). Given the diagnostic challenges in confirming immune-mediated leukopenia, we adopted classical criteria (concurrent AIHA and immune thrombocytopenia [ITP]). Secondary AIHA was defined by an established underlying disorder considered causally associated with AIHA. Patients with concurrent autoimmune thyroiditis, pernicious anemia, or undifferentiated connective tissue disease (UCTD), defined as persistent ANA ≥1:80 on at least two occasions without fulfilling established classification criteria for a definitive CTD, were not classified as secondary AIHA. Bone marrow response was assessed using the Bone Marrow Responsiveness Index (BMRI = absolute reticulocyte count × [patient Hb/normal Hb]) and Reticulocyte Production Index (RPI = [reticulocyte %/2] × [hematocrit/0.45]) (35).

### 2.3 Evaluation of treatment and outcome

Therapeutic inclusion required full-dose administration. Post-CR consolidation, cytotoxic chemotherapy, intravenous immunoglobulin [IVIG], erythropoietin [EPO], plasma exchange, immunomodulators, and traditional Chinese medicine were not regarded as standard treatment in this study. RTX was implemented as standard-dose (R-SD; 375 mg/m² weekly ×4 or 1000 mg biweekly ×2) and low-dose (R-LD; 100 mg weekly ×4). Responses were classified as complete (CR: Hb ≥12 g/dL [male] or ≥11 g/dL [female], not attributed to transfusion), partial (PR: either ≥2 g/dL Hb increase from baseline or Hb ≥10 g/dL without transfusion), or no response (NR: neither CR nor PR). No response to GCs specifically required failure to achieve PR after ≥3 weeks of prednisone (≥1 mg/kg/day). Refractory AIHA was defined as NR to ≥3 regimens, including splenectomy and/or ≥1 IST (2). Outcomes included overall survival (OS; time from diagnosis to death), event-free survival (EFS; time from diagnosis to first relapse or death), relapse (Hb decline ≥2 g/dL with evidence of active hemolysis, such as increased LDH, IBIL, or reticulocytosis, independent of blood loss, transfusions, or other causes of anemia), thrombosis, and infections (pulmonary/opportunistic).

### 2.4 Statistical analysis

All analyses were conducted using SPSS (version 27.0) and R (version 4.5.0). Missing data were handled using complete-case analysis, and no imputation was performed. Continuous variables with normal distribution were presented as mean ± standard deviation (SD) and analyzed using Student’s t-test or one-way ANOVA, while non-normally distributed data were reported as median (interquartile range, IQR) and compared via Mann-Whitney U or Kruskal-Wallis tests. Categorical variables were summarized as frequencies (%) and assessed using Pearson’s chi-square or Fisher’s exact tests. All statistical tests were two-sided, with *P* < 0.05 considered statistically significant. Time-to-event outcomes were evaluated using Kaplan-Meier analysis with 95% confidence intervals (CIs) calculated. Median follow-up duration was estimated using reverse Kaplan-Meier analysis. Univariable and multivariable Cox regression analyses were performed using the survival package in R, with verification of proportional hazards (PH) assumptions and elimination of collinear variables. Variables with P < 0.2 in univariable analysis were included in the multivariable model. Visualization was implemented with ggplot2 package.

## 3 Results

### 3.1 Clinical and laboratory profiles of cohort

The cohort comprised 566 AIHA patients (median onset age 48 years, 70% female), including 449 wAIHA and 117 mAIHA cases. Both groups showed female predominance but with earlier onset in mAIHA cases, exhibiting a distinctive bimodal age distribution (peaks at 20/60 years, trough at 40 years; **Supplementary Figure 1A**). Notably, 347 patients (61.3%) presented with severe anemia (Hb <6 g/dL) at onset. Secondary forms were more prevalent in mAIHA (39.3% vs 22.5%, **Table 1**), especially AIDs. Among secondary cases, AIDs and LPDs were the most prevalent etiologies. Systemic lupus erythematosus (SLE) constituted the majority of AIDs, whereas B-cell LPDs, particularly CD5-CD10- small B-cell LPDs, dominated the LPD-associated cases. A comprehensive catalog of all underlying secondary etiologies is detailed in **Supplementary Table 1**. Serologically, IgG with or without C3 predominated in both wAIHA and mAIHA, with IgA and/or IgM observed in 12% of wAIHA and 16.2% of mAIHA. Notably, mAIHA demonstrated a significantly higher prevalence of IgM-seropositivity compared to wAIHA. Evans syndrome (13.3%, n=75) manifested as concurrent AIHA/ITP (40%), AIHA-first presentation (36%), or ITP-first presentation (24%) (**Supplementary Figure 1B**). Laboratory analysis showed that mAIHA exhibited distinct cAIHA-like features including decreased RBC/hematocrit, elevated MCV/MCH/MCHC, and higher lymphocyte percentages, despite comparable Hb levels and hemolytic markers with wAIHA. Four patients had UGT1A1-confirmed Gilbert syndrome, which may confound the interpretation of bilirubin levels during hemolysis. Acute aplastic crisis was documented in 13 wAIHA (2.9%) and 5 mAIHA (4.3%) patients, manifesting as pancytopenia (n=4), erythroid/leukocytic reduction (n=3), and isolated erythroid hypoplasia (n=11). Both cohorts exhibited spherocytosis (42.4%, n=240) with comparable acidified glycerol 50% lysis time (75 vs. 80 seconds) and bone marrow compensation impairment (BMRI<121: 35.0%, n=198). Notably, mAIHA showed significantly impaired erythropoiesis compared to wAIHA, as evidenced by lower BMRI and RPI.

**Table 1:** Baseline demographic and clinical characteristics of cohort.

Characteristics	Overall (N = 566)	wAIHA (N = 449)	mAIHA (N = 117)	*P* value
Asian, n (%)	566 (100%)	449 (100%)	117 (100%)	-
Female, n (%)	396 (70.0%)	321 (71.5%)	75 (64.1%)	0.120
Age of onset, years†	48 (30, 61)	48 (32, 61)	43 (20, 58)	**0.006**
*Hb at onset, g/dL†	5.4 (4.3, 6.5)	5.4 (4.4, 6.5)	5.2 (4.1, 6.5)	0.690
Secondary, n (%)	147 (26.0%)	101 (22.5%)	46 (39.3%)	**< 0.001**
--AIDs	82 (55.8%)	50 (49.5%)	32 (69.6%)	**< 0.001**
--LPDs	61 (41.5%)	48 (47.5%)	13 (28.3%)	0.896
Serological type (DAT), n (%)				
--IgG	190 (33.6%)	176 (39.2%)	14 (12.0%)	**< 0.001**
--C3	18 (3.2%)	18 (4.0%)	0	0.057
--IgG+C3	285 (50.4%)	201 (44.8%)	84 (71.8%)	**< 0.001**
--IgM-mediated	32 (5.7%)	20 (4.5%)	12 (10.3%)	**0.016**
--IgA-mediated	26 (4.6%)	24 (5.3%)	2 (1.7%)	0.094
--IgM+IgA-mediated	15 (2.7%)	10 (2.2%)	5 (4.3%)	0.366
Evans syndrome, n (%)	75 (13.3%)	58 (12.9%)	17 (14.5%)	0.647
CA scores†	22 (12, 34)	14 (12, 22)	42 (40, 68)	**< 0.001**
Lymphocyte, %†	20.8 (13.6, 28.0)	19.9 (13.1, 27.8)	22.5 (14.8, 32.7)	**0.013**
RBC count, 10^12^/L†	1.8 (1.4, 2.3)	1.9 (1.4, 2.3)	1.6 (1.2, 2.2)	**0.001**
Hematocrit, % (mean ± sd)	20.4 ± 6.3	20.8 ± 5.9	19.0 ± 7.4	**0.015**
Hb, g/dL†	6.6 (5.2, 8.2)	6.7 (5.2, 8.2)	6.5 (4.9, 8.4)	0.619
MCV, fL (mean ± sd)	112.5 ± 14.9	111.3 ± 14.7	117.1 ± 15.0	**< 0.001**
MCH, pg†	36.1 (33.3, 39.4)	35.8 (33.1, 38.7)	39.3 (34.4, 48.4)	**< 0.001**
MCHC,g/L†	324 (309, 343)	322 (307, 338)	335 (316, 384)	**< 0.001**
LDH, U/L†	463 (333.1, 666.9)	471.6 (335.3, 693.6)	432 (319.6, 609.3)	0.189
IBIL, μmol/L†	43.2 (26.0, 66.4)	44.6 (26.7, 67.9)	42 (24.9, 57.2)	0.207
FHb, mg/L†	99.7 (41.3, 183)	102.4 (41.2, 182)	91.5 (42.8, 183.8)	0.949
HAP, g/L†	0.063 (0.063, 0.375)	0.063 (0.063, 0.375)	0.063 (0.063, 0.185)	0.359
Ret count, 10^9^/L†	280.4 (164.6, 392.4)	291.9 (185.5, 403.2)	200.2 (141.7, 302.6)	**< 0.001**
Ret percentage, %†	15.6 (8.7, 23.4)	17.1 (9.5, 23.3)	13.6 (7.6, 24.4)	0.215
BMRI†	157.3 (86.6, 246.7)	173.9 (96.1, 253.7)	125.3 (68.5, 195.3)	**< 0.001**
RPI†	3.4 (2.0, 5.2)	3.6 (2.2, 5.4)	2.5 (1.7, 4.1)	**0.001**
EPO, IU/L†	170.5 (81.8, 445.2)	170.3 (78.2, 443.8)	176.6 (90.1, 453.2)	0.889

*Hb at onset was defined as the lowest Hb level measured before the first documented PR/CR.

^†^denotes median (interquartile range, IQR); n (%) denotes number (percentage); sd denotes standard deviation; Defined IgM-mediated: IgM (alone or with IgG/C3); IgA-mediated: IgA (alone or with IgG/C3); IgM+IgA-mediated: IgM+IgA (± IgG/C3). Only secondary causes over 5 cases are shown.​

wAIHA/mAIHA, warm/mixed autoimmune hemolytic anemia; Hb, hemoglobin; AIDs, autoimmune and autoinflammatory diseases; LPDs, lymphoproliferative diseases; DAT, direct antiglobulin test; Ig, immunoglobulin; C3: complement component 3; CA, cold agglutinin; RBC, red blood cell; MCV, mean corpuscular volume; MCH, mean corpuscular hemoglobin; MCHC, mean corpuscular hemoglobin concentration; LDH, lactate dehydrogenase; IBIL, indirect bilirubin; FHb, free hemoglobin; HAP, haptoglobin; Ret, reticulocyte; BMRI, bone marrow responsiveness index; RPI, reticulocyte production index; EPO, erythropoietin.

Bold values indicate statistical significance (P < 0.05).

### 3.2 Treatment landscape and response

GCs remained the first-line therapy in 556 patients (556/566, 98.2%). 92.3% of wAIHA patients and 97.3% of mAIHA patients achieved a hematologic response. The remaining 10 patients were initially treated with RTX (n=4), CTX (n=1) or splenic artery embolization (n=1) due to contraindications to GCs, chemotherapy (n=2) due to primary LPDs, or remained untreated (n=2). Second-line therapy was implemented in 365 cases, predominantly RTX (n=199), which yielded comparable observed ORRs between the SD and LD regimens (86.4% vs. 89.0%). CsA (n=64) was also frequently adopted as second-line treatment, followed by androgens (n=48), CTX (n=17), MMF (n=11), splenectomy (n=9), and BTK inhibitors (n=5). 153 patients (27.0%) received later-line therapy (≥3 lines), including 14 (3.1%) refractory cases with wAIHA and 4 (3.4%) with mAIHA. IVIG (n=213), EPO (n=34), plasma exchange (n=19), and roxadustat (n=8) were used as adjunctive therapies in some patients. One multi-refractory patient who had received up to 13 lines of therapy achieved CR with BCMA-CD3 bispecific antibody therapy (28). The heterogeneous therapeutic landscape in this cohort is delineated in **Figure 1A**. To eliminate confounding factors from underlying diseases, therapeutic responses were analyzed separately (**Table 2**). Outcomes in the primary cohort may serve as a more objective reference for regimen efficacy. Responses for regimens with ≥5 evaluable cases are delineated in **Figure 1B** (primary AIHA) and **Figure 1C** (secondary AIHA).

**Figure 1:**
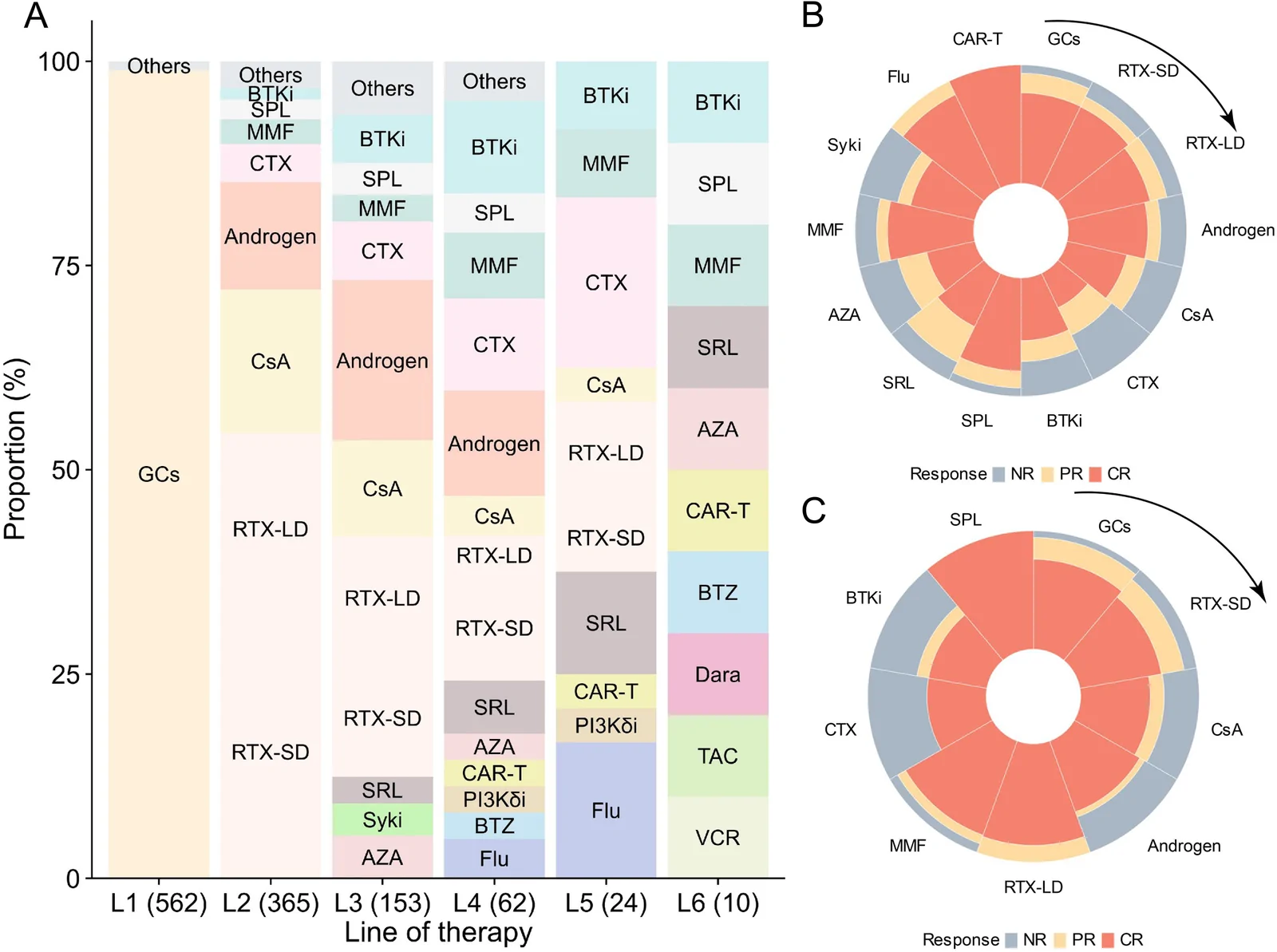
Therapeutic landscape and response assessment. **(A)** Distribution of therapeutic regimens across sequential lines of therapy (L1–L6), illustrating the heterogeneity of treatment patterns from first-line to refractory settings. **(B, C)** Evaluation of therapeutic efficacy in **(B)** primary and **(C)** secondary AIHA. Regimens are arranged clockwise starting from GCs (indicated by the arrow), ranked by frequency of use. Stacked bars represent the proportion of complete response (CR), partial response (PR), and no response (NR) for each regimen. Detailed response rates and proportions are provided in **Table 2**. GCs, glucocorticoids; RTX, rituximab (SD, standard dose; LD, low dose); CsA, cyclosporine A; CTX, cyclophosphamide; MMF, mycophenolate mofetil; AZA, azathioprine; SPL, splenectomy; BTKi, Bruton tyrosine kinase inhibitor; Syki, spleen tyrosine kinase inhibitor; SRL, sirolimus; BTZ, bortezomib; Dara, daratumumab; PI3Kδi, phosphatidylinositol 3-kinase delta inhibitor; CAR-T, chimeric antigen receptor T-cell therapy; Flu, fludarabine; TAC, tacrolimus; VCR, vincristine. Others include splenic artery embolization, B-cell activating factor receptor (BAFF-R) inhibitors, bendamustine, thalidomide, etc.

**Table 2:** Therapeutic responses of various regimens in primary and secondary AIHA.

Treatment regimens	Total		Primary AIHA		Secondary AIHA	
	n	ORR/CR, %	n	ORR/CR, %	n	ORR/CR, %
Glucocorticoids*	552	93.3/73.4	410	92.9/73.7	142	94.4/72.5
Rituximab (Standard dose)‡	145	86.4/72.1	108	85.5/73.6	37	89.2/67.6
Rituximab (Low dose)‡	115	89.0/74.4	94	86.5/71.9	21	100.0/85.7
Cyclosporine A	87	69.0/52.9	60	68.3/51.7	27	70.4/55.6
Androgens†	86	75.6/66.3	64	78.1/67.2	22	68.2/63.6
Cyclophosphamide	41	56.1/36.6	31	58.1/32.3	10	50.0/50.0
BTK Inhibitors	27	66.7/51.8	17	70.6/52.9	10	60.0/50.0
Mycophenolate Mofetil	24	87.5/79.1	11	81.8/72.7	13	92.3/84.6
Splenectomy	19	94.8/84.2	14	92.9/78.6	5	100.0/100.0
Sirolimus	15	86.6/53.3	12	83.3/50.0	3	100.0/66.7
Azathioprine	15	66.7/46.7	12	66.7/41.7	3	66.7/66.7
Syk Inhibitors(20)	10	70.0/60.0	9	66.7/55.6	1	100.0/100.0
Fludarabine (+Rituximab)	10	90.0/80.0	8	100.0/87.5	2	50.0/50.0
CAR-T Therapy(27)	6	100.0/100.0	6	100.0/100.0	—	—

*Four patients who received GCs as initial therapy were unassessable for therapeutic response.

^‡^Rituximab standard dose: 375 mg/m² weekly for 4 doses or 1000 mg every 2 weeks for 2 doses; low dose: 100 mg weekly for 4 doses.

^†^Androgens are often used in combination with other agents.

AIHA, autoimmune hemolytic anemia; ORR, overall response rate; CR, complete response; BTK, Bruton tyrosine kinase; Syk, spleen tyrosine kinase; CAR-T, chimeric antigen receptor T-cell.

### 3.3 Relapses and thrombotic/infectious complications

Among patients who experienced a relapse, the median duration from diagnosis to the first relapse was 8.0 months overall, with 8.0 months in wAIHA and 9.5 months in mAIHA. With a median EFS of 17 months, the estimated 1-/3-/5-year EFS rates were 54.5%/37.5%/29.9% respectively (**Figure 2A**). In patients with >6-month follow-up, relapses occurring ≤6 months post-diagnosis showed significantly inferior OS versus those >6 months (HR 2.04, p=0.021) (**Figure 2B**). Analysis of 365 relapse events (multiple triggers permitted) identified drug reduction/cessation (71.8%), infections (27.1%, predominantly respiratory), physiological stressors (4.9%: exertion, trauma, surgery, pregnancy, psychological stress), and underlying disease flares (1.9%) as the contributing factors. Thrombosis occurred in 46 (10.2%) wAIHA and 9 (7.7%) mAIHA patients, with one patient experiencing both arterial and venous thrombosis. Arterial events (n=28) predominantly involved stroke (n=19) and the splenic artery (n=8), while venous thrombosis (n=28) primarily manifested as lower extremity deep vein thrombosis (left unilateral n=9, bilateral n=6, right unilateral n=5) and pulmonary embolism (n=5). Notably, 81.8% (45/55) of patients experienced thrombosis during active hemolysis, supporting a prothrombotic state associated with active hemolysis. The early disease phase appeared to represent a high-risk window, with 43.6% (24/55) of patients having thrombosis identified within one month after diagnosis. Concomitant hypercoagulable risk factors were identified in a significant subset of patients (27.3%, 15/55). Notably, some patients exhibited overlapping risk profiles. The identified factors included antiphospholipid antibody positivity (n=8), confirmed antiphospholipid syndrome (n=3), prior splenectomy (n=3), and protein C/S deficiency (n=3). Furthermore, Fine-Gray competing risk modeling quantified the cumulative incidence of relapse and thrombotic events (**Figures 2C, D**, respectively), with the 1-, 3-, and 5-year cumulative incidences of thrombosis reaching 6.51%, 8.42%, and 9.31%. The immunocompromised state induced by AIHA and its therapeutic interventions predisposed patients to significant infectious risks, particularly pulmonary (33.6% wAIHA [151/449] vs 34.2% mAIHA [40/117]) and opportunistic infections (12.7% [57/449] vs 12.8% [15/117]). Fungal pathogens constituted the predominant opportunistic microorganisms (n=58, 80.6%), with identified species comprising *Aspergillus* (n=11, 15.3%), *Candida* (n=11, 15.3%), *Pneumocystis jirovecii* (n=9, 12.5%), and *Cryptococcus* (n=4, 5.6%). Bacterial and viral pathogens included *Mycobacterium tuberculosis* (n=14, 19.4%), varicella-zoster virus (n=7, 9.7%), parvovirus B19 (n=4, 5.6%), and other microorganisms (*Nocardia*, *Pseudomonas aeruginosa*, *Acinetobacter baumannii*). Notably, polymicrobial infection patterns were frequently observed, reflecting profound immunosuppression.

**Figure 2:**
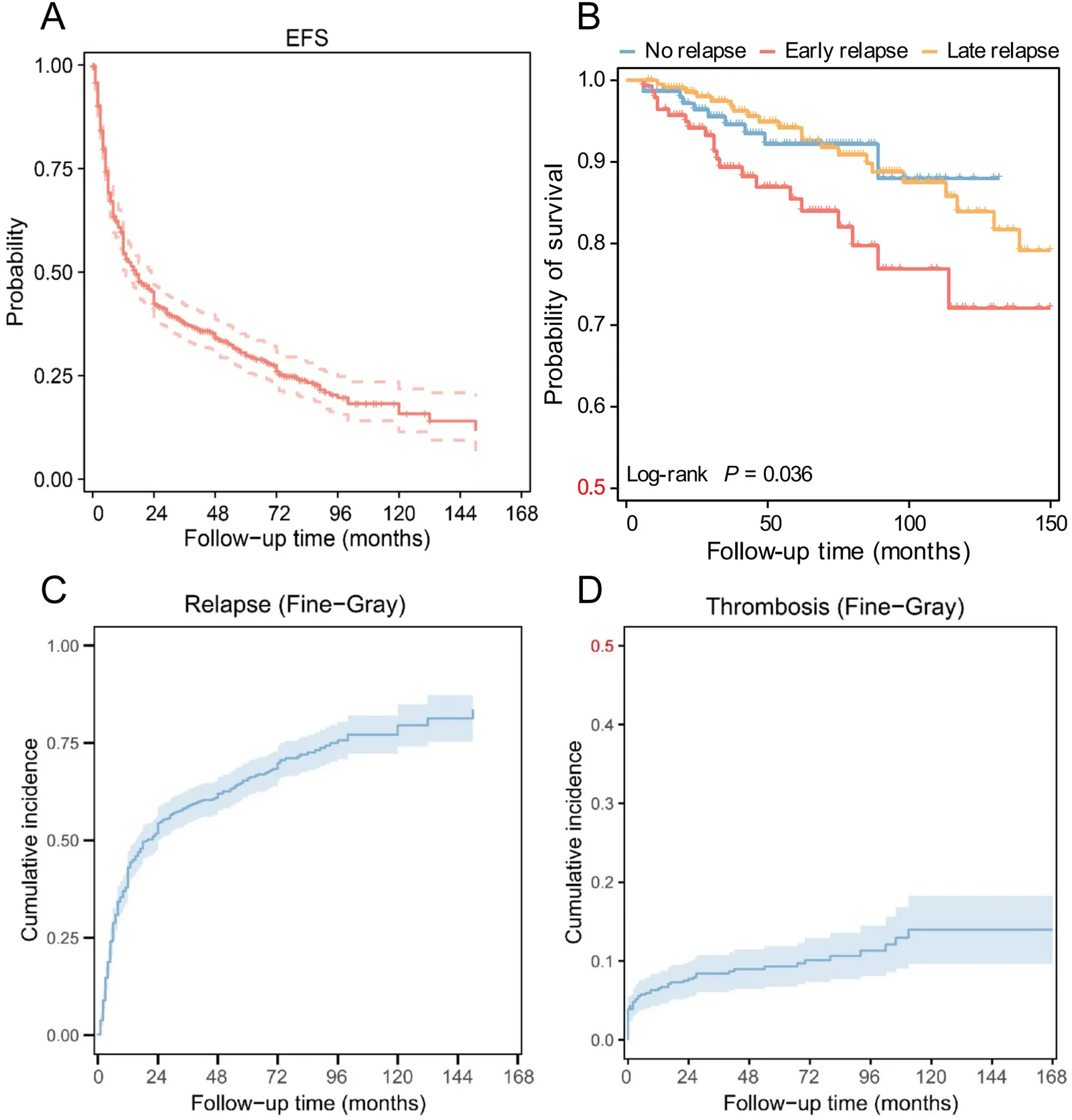
Event-free survival and cumulative incidence of complications. **(A)** Kaplan-Meier estimates of event-free survival (EFS) for the entire cohort. **(B)** OS stratified by the timing of relapse (≤6 months vs. >6 months vs. relapse-free). **(C, D)** Cumulative incidence functions for **(C)** relapse and **(D)** thrombotic events, estimated using the Fine-Gray model with death as a competing risk. OS, overall survival; EFS, event-free survival.

### 3.4 Survival analysis of overall survival

In the entire cohort (median follow-up 57 months for wAIHA and 59 months for mAIHA), the estimated 1-, 3- and 5-year OS rates were 96%, 92% and 89% (**Figure 3A**). As of the final unified follow-up, 475 patients (372 wAIHA and 103 mAIHA) were successfully evaluated, among whom the CR rates at the final assessment were 70.4% and 72.8%, with mortality rates of 16.1% (60/372) and 16.5% (17/103), respectively. Analysis of 66 deaths with identifiable causes (51 wAIHA and 15 mAIHA) revealed that 21 (41.2%) and 9 (60.0%) occurred with active AIHA, including 13 (25.5%) and 4 (26.7%) directly attributed to AIHA progression. Infections predominated non-AIHA deaths (31.8%, 21/66; including pulmonary [n=17], fungal [n=9], and sepsis [n=4]). Other causes included cerebro/cardiovascular events, non-LPD malignancies, and multiple organ failure (4 each), plus LPDs and renal failure (2 each). Univariable Cox analysis identified significant OS associations with IgM+IgA-mediated (IgM+IgA [± IgG/C3]; reference: IgG+C3), age ≥60 years, Hb <6 g/dL, BMRI <121, male sex, and RPI <2.3 (ranked by hazard ratio [HR]). No significant correlations were observed with CA titers (wAIHA/mAIHA), etiology (primary/secondary), ES, or spherocytosis (**Figures 4A**, **3B, C**). For detailed visualization, the corresponding Kaplan-Meier curves are presented in **Supplementary Figures 2A–H**. Given the median CA titer of 64 in mAIHA cohort, we restratified patients by titers (≥1024 vs ≤512) to assess its prognostic impact. While the high-titer group (n=28) showed a trend toward inferior OS, the results were not statistically significant (**Figure 3D**). Multivariable Cox analysis identified age ≥60 years (HR = 3.40), Hb <6 g/dL (HR = 2.23), BMRI <121 (HR = 2.04), male sex (HR = 1.82), and IgM+IgA-mediated serological pattern (HR = 3.42, versus IgG+C3) as independent risk factors associated with poorer OS (**Figure 4B**). Additional Cox analyses were performed separately for wAIHA and mAIHA subgroups (**Supplementary Figures 3**, **4**). In wAIHA, IgM-mediated cases were associated with poorer OS in univariable analysis (HR = 3.18), although this association was attenuated after multivariable adjustment (HR = 2.80, P = 0.065). In mAIHA, age ≥60 years was the only factor remaining significant in the multivariable model (HR = 4.64).

**Figure 3:**
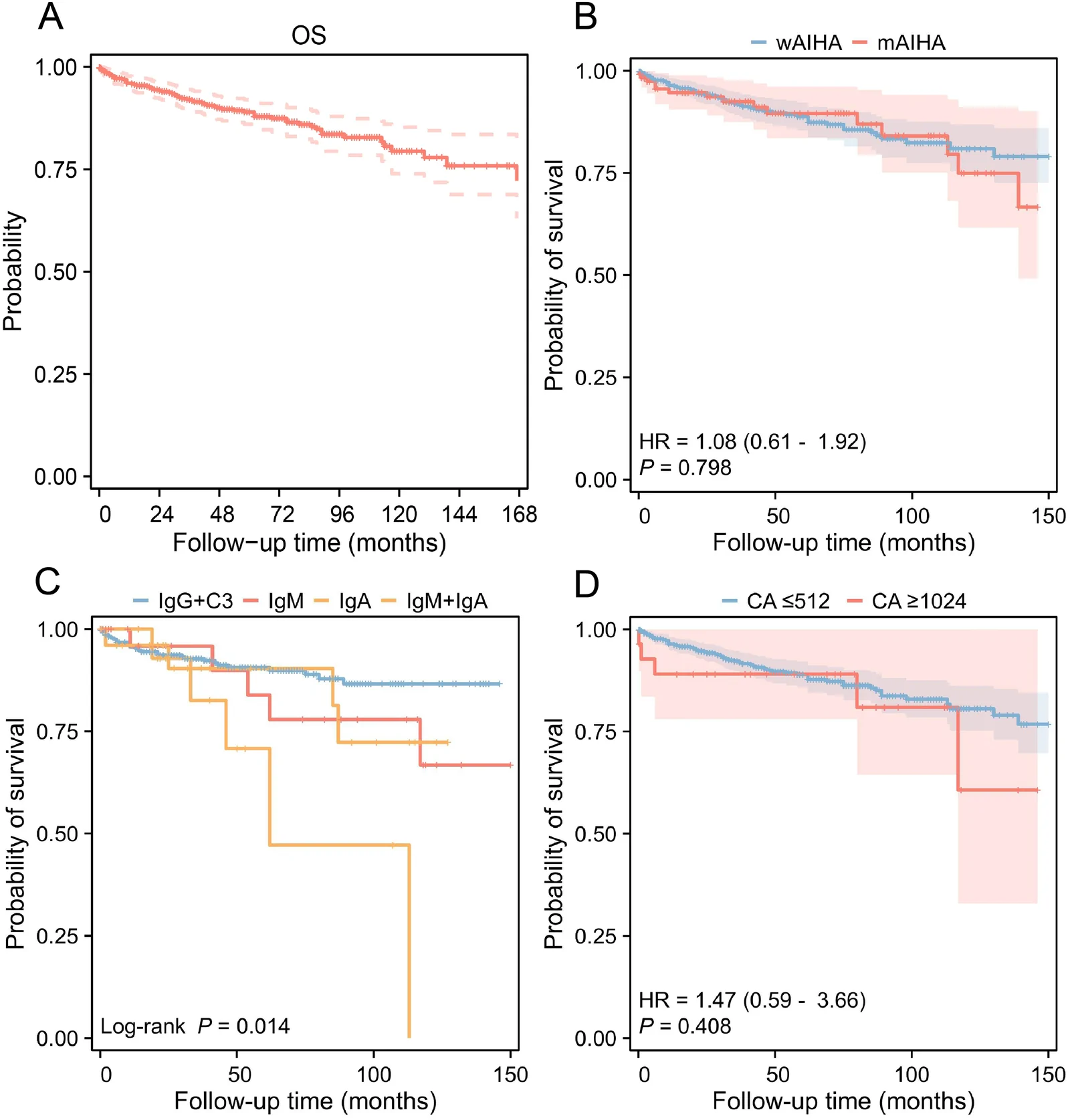
Overall survival outcomes according to clinical and serological characteristics. **(A)** Kaplan-Meier estimates of overall survival (OS) for the entire cohort. **(B, C)** Stratified OS comparisons based on: **(B)** clinical subtype (wAIHA vs. mAIHA); and **(C)** antibody mediation pattern. **(D)** OS stratified by cold agglutinin titers (≥1024 vs. ≤512). OS, overall survival; wAIHA, warm autoimmune hemolytic anemia; mAIHA, mixed autoimmune hemolytic anemia; CA, cold agglutinin.

**Figure 4:**
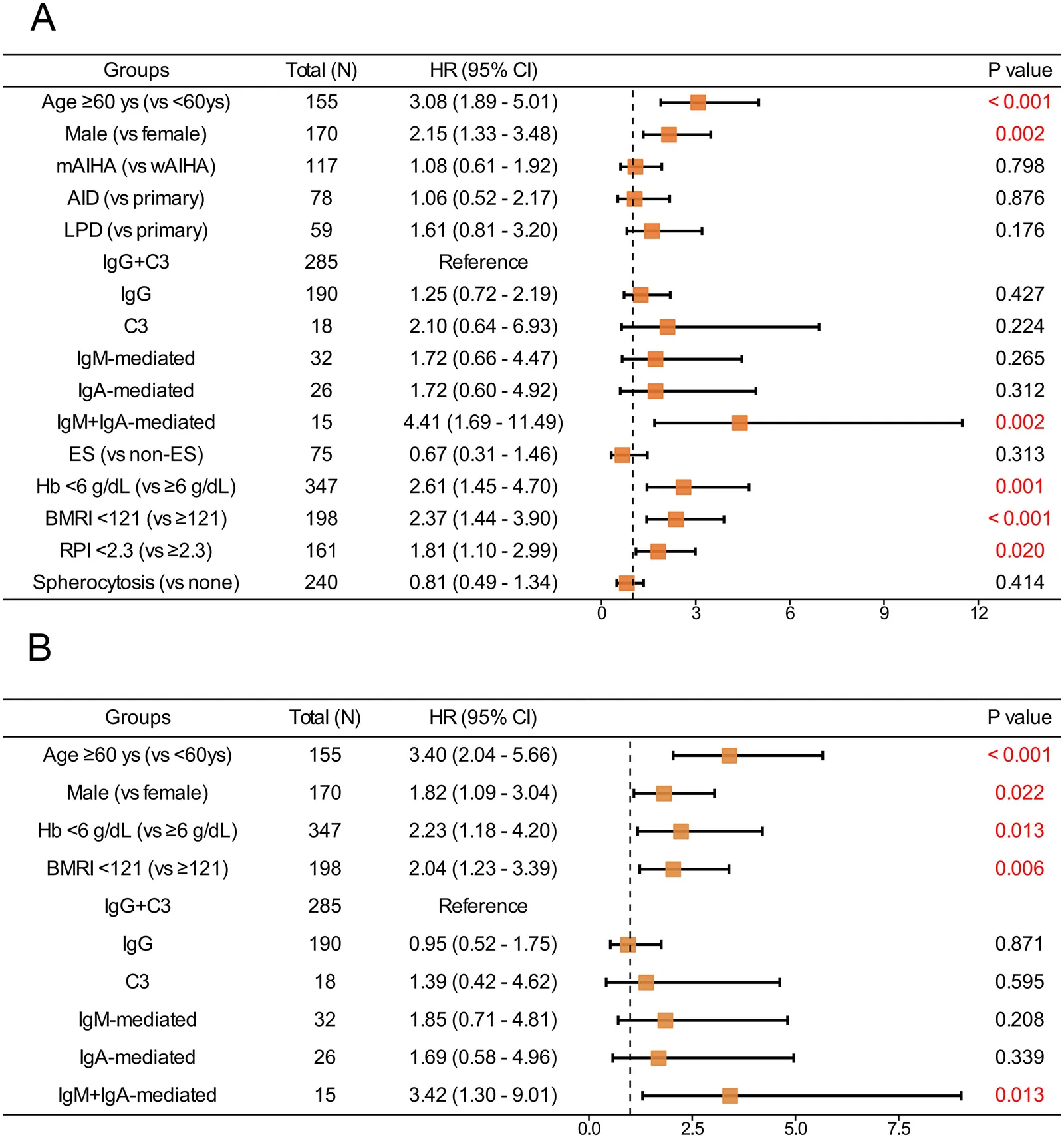
Forest plots of Cox regression analyses for overall survival (OS). **(A)** Univariable analysis identifying prognostic factors; predictors with statistical significance are highlighted in red. **(B)** Multivariable analysis. Of note, the reticulocyte production index (RPI) was excluded due to collinearity with the bone marrow responsiveness index (BMRI). The proportional hazards assumption was validated for all variables included. Abbreviations and definitions: HR, hazard ratio; CI, confidence interval; wAIHA/mAIHA, warm/mixed autoimmune hemolytic anemia; AID, autoimmune/autoinflammatory disease; LPD, lymphoproliferative disease; Ig, immunoglobulin; C3, complement component 3; IgM-mediated, IgM alone or with IgG/C3; IgA-mediated, IgA alone or with IgG/C3; IgM+IgA-mediated, IgM+IgA ± IgG/C3; ES, Evans syndrome; Hb, hemoglobin at onset; BMRI, bone marrow responsiveness index; RPI, reticulocyte production index; Spherocytosis, diagnosed by erythrocyte osmotic fragility testing (hemolysis thresholds: ≥0.52% NaCl [onset], ≥0.42% [complete]) and peripheral blood smear.

## 4 Discussion

Accounting for the majority of acquired hemolytic anemia, AIHA poses a considerable disease burden, with estimated incidence of 1.8–3 per 100,000 person-years and prevalence of approximately 17 per 100,000 persons (36, 37). While the field of AIHA is evolving rapidly, large-scale retrospective characterizations of Asian cohorts have remained notably absent. Notably, of the 629 hospitalized AIHA patients screened, 32 (5.1%) were classified as cAIHA, lower than previously reported (15–25%), possibly reflecting geographic and climatic variation. Addressing this critical gap as the largest retrospective AIHA study in Asia and the most extensive mAIHA cohort to date, our work provides a panoramic view of the clinical landscape in China, systematically investigating disease profiles, therapeutic responses, and complication patterns in 566 patients over a median 57-month follow-up.

Clinically, attention should be paid to identifying the etiology of AIHA. In our study, we established secondary forms in 22.5% wAIHA and 39.3% mAIHA cases, rather than the equal distribution often cited (1, 16). This discrepancy reflects several real-world complexities. First, no hospitalized patients with a well-established diagnosis of infection- or drug- induced AIHA were identified in this cohort, as establishing definitive causality is challenging and hemolytic episodes can be transient. Notably, pernicious anemia, autoimmune thyroiditis, and UCTD were not considered secondary causes of AIHA, as they likely reflect predisposing genetic or immunologic backgrounds rather than causative relationships (1). Beyond these, difficulties also arose from delayed recognition of underlying disorders when AIHA was the initial presentation, and the masking effect of AIHA therapies on underlying etiologies. Moreover, this suggests that mAIHA, especially, may harbor underlying secondary causes, and particularly AIDs.

Consistent with prior studies of wAIHA, our cohort exhibited female and elderly predominance. In contrast to Western populations, we observed a higher female proportion (71.5% vs. 55%) and younger age (48 vs. 58 years) (3, 37). This disparity likely stems from the distinct landscape of etiologies within our cohort, where AIDs, especially SLE, are slightly more prevalent. This contrasts with Western cohorts, where LPDs, particularly CLL (38), are more commonly implicated (39). Our cohort instead found MZL and LPL/WM as the most common LPDs. Notably, male sex was independently associated with poorer OS, aligning with a large US national AIHA cohort reporting higher in-hospital mortality and several adverse outcomes among male patients (40). While overall secondary AIHA rates were comparable between sexes, their underlying etiologies differed markedly. Secondary cases in males were predominantly LPD-associated (68.4% vs. 32.1% in females), while female cases were AID-associated (63.3% vs. 26.3% in males). This disparity may contribute to survival differences. Nevertheless, potential residual confounding makes male sex an exploratory prognostic factor necessitating further validation. Studies have suggested that IgM-mediated wAIHA typically follows a severe and fulminant clinical course (1, 41), a finding corroborated by our data (HR = 2.80, P = 0.065). Here we made the further observation that wAIHA patients with concurrent IgA involvement (IgM+IgA-mediated) may experience even poorer outcomes (HR = 4.13), though this finding requires cautious interpretation given the limited number of patients with this uncommon serological pattern. Thus, screening for IgM/IgA autoantibodies is essential in DAT-negative patients presenting with severe hemolysis to prevent misdiagnosis.

We also looked into the temporal relationship between AIHA and ITP in ES. Consistent with prior reports (42), concurrent diagnosis remained the most common pattern, yet we further observed a higher frequency of AIHA preceding ITP in sequential cases. Current comprehension of mAIHA remains elusive due to different diagnostic criteria and inadequate research (29). In our study, mAIHA was established using a broader operational definition. Because thermal amplitude was not routinely assessed, this approach may introduce phenotypic heterogeneity (33). Aligning with a prior systematic review, our study confirmed mAIHA’s unique features including predominant AIDs involvement among secondary factors and earlier onset (43 vs. 45 years) (29). Our cohort revealed a distinctive bimodal age distribution, peaking at 20 years (AIDs -related) and 60 years (LPDs -related), which is indicative of its pronounced secondary etiology. Notably, mAIHA demonstrates distinct cold agglutination features compared to wAIHA, with higher prevalence of IgM-mediated cases. IgM pentamers induce RBC aggregation (16), resulting in decreased RBC counts alongside elevated MCV, MCH, and MCHC despite relative reticulocytopenia, reflecting compromised bone marrow compensation. Furthermore, we observed no significant differences in OS, hemolytic parameters, or GCs response among patients with varying CA titers, implying that isolated CA titers might not serve as a dependable indicator of either clinical severity or treatment efficacy (29).

Despite being classified as a “benign” condition, AIHA significantly compromises quality of life through relapse, infections, and thromboembolic events. The 17-month median EFS contrasts with favorable OS. Relapse within ≤6 months significantly worsens prognosis, with drug tapering and infections being primary triggers, underscoring the significance of treatment adherence, proper tapering education, and infection monitoring. Studies suggest that vaccination against respiratory pathogens is recommended to prevent complement activation and subsequent AIHA relapses (1). Thrombotic events were frequently identified early in the disease course, with nearly 50% detected within the first month after diagnosis. Given their strong association with active hemolysis, consideration of prophylactic anticoagulation during active hemolysis may be warranted, especially for patients with identifiable risk factors. Infections, particularly opportunistic infections, significantly compromise AIHA outcomes. Our cohort identified infection as the leading direct cause of non-AIHA related mortality. This aligns with a French study that reported 12.5% 30-day mortality following infection-related hospitalization, highlighting a profound clinical dilemma that requires balancing intensive immunosuppressive therapy with infection risk management (43).

The evolving therapeutic paradigm for LPDs and AIDs has transformed AIHA management. It is important to note that our outcomes should be interpreted cautiously. First, novel agents like Syk inhibitors were preferentially administered in later-line or refractory settings (20), potentially limiting generalizability. Second, the use of concurrent therapies may obscure the independent contributions of individual agents. For instance, the reported 78.1% ORR for androgens (consistent with prior studies (44)) likely reflects combined efficacy with GCs, as androgen is typically employed as an adjunctive therapy. Furthermore, observed responses represent initial achievement rather than sustained remission. Given the shared pathophysiology between AIHA and ITP as immune-mediated cytopenias, therapeutic strategies often provide mutual reference. In ITP, single-dose rituximab (375 mg/m²) has demonstrated comparable efficacy to R-LD in treating GCs-resistant or relapsed cases (45). The observed ORRs were numerically similar between R-LD and R-SD, though the lack of a rigorous design precludes formal non-inferiority conclusions. As the early integration of RTX remains a crucial therapeutic option for severe cases, prospective studies optimizing dosing and frequency are essential to enhance medical economic efficiency. Of particular interest in refractory AIHA is the emerging role of cellular immunotherapy. While limited by a small sample size, autologous CD19 CAR T-cell therapy facilitated complete and treatment-free remission in our multi-refractory cases. Notably, the use of BCMA-CD3 bispecific antibody therapy (T-cell engagers) as a salvage strategy in two relapsed cases demonstrated preliminary efficacy, highlighting a promising approach that merits prospective validation.

These limitations and evolving therapies underscore the need for rigorously designed prospective trials assessing not only response rates but also EFS and quality of life. In summary, this large retrospective study delineates the clinical landscape of AIHA in Chinese patients, with particular focus on the distinct features of mAIHA. Our findings bridge critical knowledge gaps in Asian AIHA management by identifying prognostic indicators and offering evidence-based guidance for clinical decision-making and the development of novel therapies.

## Data Availability

The data supporting the conclusions of this article will be made available by the authors upon reasonable request and without undue reservation.

## References

[1] Michel M Crickx E Fattizzo B Barcellini W . Autoimmune haemolytic anaemias. Nat Rev Dis Primers. (2024) 10:82. doi: 10.1038/s41572-024-00566-2 39487134

[2] Jäger U Barcellini W Broome CM Gertz MA Hill A Hill QA . Diagnosis and treatment of autoimmune hemolytic anemia in adults: recommendations from the first international consensus meeting. Blood Rev. (2020) 41:100648. doi: 10.1016/j.blre.2019.100648 31839434

[3] Barcellini W Fattizzo B Zaninoni A Radice T Nichele I Di Bona E . Clinical heterogeneity and predictors of outcome in primary autoimmune hemolytic anemia: a GIMEMA study of 308 patients. Blood. (2014) 124:2930–6. doi: 10.1182/blood-2014-06-583021 25232059

[4] Schär DT Daskalakis M Mansouri B Rovo A Zeerleder S . Thromboembolic complications in autoimmune hemolytic anemia: retrospective study. Eur J Haematol. (2022) 108:45–51. doi: 10.1111/ejh.13710 34551149 PMC9297871

[5] Fattizzo B Barcellini W . Autoimmune hemolytic anemia: causes and consequences. Expert Rev Clin Immunol. (2022) 18:731–45. doi: 10.1080/1744666X.2022.2089115 35702053

[6] Hill QA Hill A Berentsen S . Defining autoimmune hemolytic anemia: a systematic review of the terminology used for diagnosis and treatment. Blood Adv. (2019) 3:1897–906. doi: 10.1182/bloodadvances.2019000036 31235526 PMC6595261

[7] Barcellini W Fattizzo B . Autoimmune hemolytic anemias: challenges in diagnosis and therapy. Transfus Med Hemother. (2024) 51:321–31. doi: 10.1159/000540475 39371250 PMC11452171

[8] Gehrs BC Friedberg RC . Autoimmune hemolytic anemia. Am J Hematol. (2002) 69:258–71. doi: 10.1002/ajh.10062 11921020

[9] Ward FJ Hall AM Cairns LS Leggat AS Urbaniak SJ Vickers MA . Clonal regulatory T cells specific for a red blood cell autoantigen in human autoimmune hemolytic anemia. Blood. (2008) 111:680–7. doi: 10.1182/blood-2007-07-101345 17761830 PMC2575838

[10] Ciudad M Ouandji S Lamarthée B Cladière C Ghesquière T Nivet M . Regulatory T-cell dysfunctions are associated with increase in tumor necrosis factor α in autoimmune hemolytic anemia and participate in Th17 polarization. Haematologica. (2024) 109:444–57. doi: 10.3324/haematol.2023.282859 37534543 PMC10828774

[11] Barcellini W Zaninoni A Giannotta JA Fattizzo B . New insights in autoimmune hemolytic anemia: from pathogenesis to therapy stage 1. J Clin Med. (2020) 9:3859. doi: 10.3390/jcm9123859 33261023 PMC7759854

[12] Iwamoto S Kamesaki T Oyamada T Okuda H Kumada M Omi T . Reactivity of autoantibodies of autoimmune hemolytic anemia with recombinant rhesus blood group antigens or anion transporter band3. Am J Hematol. (2001) 68:106–14. doi: 10.1002/ajh.1161 11559950

[13] Brodsky RA . Warm autoimmune hemolytic anemia. N Engl J Med. (2019) 381:647–54. doi: 10.1056/NEJMcp1900554 31412178

[14] Roumier M Loustau V Guillaud C Languille L Mahevas M Khellaf M . Characteristics and outcome of warm autoimmune hemolytic anemia in adults: new insights based on a single-center experience with 60 patients. Am J Hematol. (2014) 89:E150–5. doi: 10.1002/ajh.23767 24847759

[15] Damelang T de Taeye SW Rentenaar R Roya-Kouchaki K de Boer E Derksen NIL . The influence of human IgG subclass and allotype on complement activation. J Immunol. (2023) 211:1725–35. doi: 10.4049/jimmunol.2300307 37843500 PMC10656437

[16] Berentsen S Barcellini W . Autoimmune hemolytic anemias. N Engl J Med. (2021) 385:1407–19. doi: 10.1056/NEJMra2033982 34614331

[17] Berentsen S Fattizzo B Barcellini W . The choice of new treatments in autoimmune hemolytic anemia: how to pick from the basket? Front Immunol. (2023) 14:1180509. doi: 10.3389/fimmu.2023.1180509 37168855 PMC10165002

[18] Michalak SS Olewicz-Gawlik A Rupa-Matysek J Wolny-Rokicka E Nowakowska E Gil L . Autoimmune hemolytic anemia: current knowledge and perspectives. Immun Ageing. (2020) 17:38. doi: 10.1186/s12979-020-00208-7 33292368 PMC7677104

[19] Nikitin E Kislova M Morozov D Belyakova V Suvorova A Sveshnikova J . Ibrutinib in combination with rituximab is highly effective in treatment of chronic lymphocytic leukemia patients with steroid refractory and relapsed autoimmune cytopenias. Leukemia. (2023) 37:1464–73. doi: 10.1038/s41375-023-01891-3 37202442 PMC10195665

[20] Zhao X Sun J Zhang Z Chen M Gong T He G . Sovleplenib in patients with primary or secondary warm autoimmune haemolytic anaemia: results from phase 2 of a randomised, double-blind, placebo-controlled, phase 2/3 study. Lancet Haematol. (2025) 12:e97–e108. doi: 10.1016/S2352-3026(24)00344-2 39799953

[21] Kuter DJ Piatek C Röth A Siddiqui A Numerof RP Dummer W . Fostamatinib for warm antibody autoimmune hemolytic anemia: phase 3, randomized, double-blind, placebo-controlled, global study (FORWARD). Am J Hematol. (2024) 99:79–87. doi: 10.1002/ajh.27144 37929318

[22] Noto A Cassin R Mattiello V Reda G . The role of novel agents in treating CLL-associated autoimmune hemolytic anemia. J Clin Med. (2021) 10:2064. doi: 10.3390/jcm10102064 34065833 PMC8151128

[23] McGlothlin J Abeykoon J Al-Hattab E Ashrani AA Elliott M Hook CC . Bortezomib and daratumumab in refractory autoimmune hemolytic anemia. Am J Hematol. (2023) 98:E263–5. doi: 10.1002/ajh.27025 37417424

[24] Acar SO Tahta N Al IO Erdem M Gözmen S Karapınar TH . Sirolimus is effective and safe in childhood relapsed-refractory autoimmune cytopenias: a multicentre study. Scand J Immunol. (2024) 100:e13376. doi: 10.1111/sji.13376 38741164

[25] Cheekati M Murakhovskaya I . Anti-B-cell-activating factor (BAFF) therapy: a novel addition to autoimmune disease management and potential for immunomodulatory therapy in warm autoimmune hemolytic anemia. Biomedicines. (2024) 12:1597. doi: 10.3390/biomedicines12071597 39062171 PMC11275058

[26] Barcellini W Pane F Patriarca A Murakhovskaya I Terriou L DeSancho MT . Parsaclisib for the treatment of primary autoimmune hemolytic anemia: results from a phase 2, open‐label study. Am J Hematol. (2024) 99:2313–20. doi: 10.1002/ajh.27493 39435908

[27] Li R Pan H Zhang L Ma J Li W Gao Z . CD19 CAR T-cell therapy for autoimmune hemolytic anemia. N Engl J Med. (2026) 394:253–67. doi: 10.1056/NEJMoa2509820 41534043

[28] Zhang L Gao Z Pan H Li R Fang L Li W . BCMA-targeted T-cell engager for autoimmune hemolytic anemia after CD19 CAR T-cell therapy. N Engl J Med. (2025) 392:2282–4. doi: 10.1056/NEJMc2502297 40499177

[29] Jacobs JW Raza S Clark LM Stephens LD Allen ES Woo JS . Mixed autoimmune hemolytic anemia: a systematic review of epidemiology, clinical characteristics, therapies, and outcomes. Am J Hematol. (2025) 100:1397–407. doi: 10.1002/ajh.27721 40392014 PMC12232602

[30] Versino F Markovic U Pedone GL Pergolizzi S Passamonti F Barcellini W . Mixed type autoimmune hemolytic anemia: clinical severity and treatment patterns in a large observational study. Am J Hematol. (2026) 101:1650–3. doi: 10.1002/ajh.70321 41998867

[31] Barcellini W Zaninoni A Fattizzo B Giannotta JA Lunghi M Ferrari A . Predictors of refractoriness to therapy and healthcare resource utilization in 378 patients with primary autoimmune hemolytic anemia from eight italian reference centers. Am J Hematol. (2018) 93:E243–6. doi: 10.1002/ajh.25212 29981267

[32] Cherif H Cai Q Crivera C Leon A Rahman I Leval A . Overall survival and treatment patterns among patients with warm autoimmune hemolytic anemia in Sweden: a nationwide population‐based study. Eur J Haematol. (2025) 114:129–38. doi: 10.1111/ejh.14311 39382375 PMC11613582

[33] Loriamini M Cserti-Gazdewich C Branch DR . Autoimmune hemolytic anemias: classifications, pathophysiology, diagnoses and management. Int J Mol Sci. (2024) 25:4296. doi: 10.3390/ijms25084296 38673882 PMC11049952

[34] Fattizzo B Marchetti M Michel M Cantoni S Frederiksen H Giordano G . Diagnosis and management of evans syndrome in adults: first consensus recommendations. Lancet Haematol. (2024) 11:e617–28. doi: 10.1016/S2352-3026(24)00144-3 38968944

[35] Hevessy Z Toth G Antal-Szalmas P Tokes-Fuzesi M Kappelmayer J Karai B . Algorithm of differential diagnosis of anemia involving laboratory medicine specialists to advance diagnostic excellence. Clin Chem Lab Med. (2024) 62:410–20. doi: 10.1515/cclm-2023-0807 37823455

[36] Hansen DL Möller S Andersen K Gaist D Frederiksen H . Increasing incidence and prevalence of acquired hemolytic anemias in Denmark, 1980-2016. Clin Epidemiol. (2020) 12:497–508. doi: 10.2147/CLEP.S250250 32547241 PMC7250289

[37] Maquet J Lafaurie M Walter O Sailler L Sommet A Lapeyre-Mestre M . Epidemiology of autoimmune hemolytic anemia: a nationwide population-based study in France. Am J Hematol. (2021) 96:E291–3. doi: 10.1002/ajh.26213 33930203

[38] Visco C Barcellini W Maura F Neri A Cortelezzi A Rodeghiero F . Autoimmune cytopenias in chronic lymphocytic leukemia. Am J Hematol. (2014) 89:1055–62. doi: 10.1002/ajh.23785 24912821

[39] Xing L Zhao M Wang Y Feng Y Qu Y Duan N . Characteristics of patients with autoimmune haemolytic anaemia secondary to lymphoproliferative disorder: a single-centre retrospective analysis. Sci Rep. (2019) 9:19716. doi: 10.1038/s41598-019-56162-y 31873137 PMC6928151

[40] Abdelhay A Jain T Srivastava A Avalos Sugastti E Mostafa M Reghis M . Gender disparities in outcomes of autoimmune hemolytic anemia admissions: an analysis of the National Inpatient Sample. Blood. (2023) 142:5197. doi: 10.1182/blood-2023-178263

[41] Arndt PA Leger RM Garratty G . Serologic findings in autoimmune hemolytic anemia associated with immunoglobulin M warm autoantibodies. Transfusion. (2009) 49:235–42. doi: 10.1111/j.1537-2995.2008.01957.x 18980619

[42] Michel M Chanet V Dechartres A Morin A-S Piette J-C Cirasino L . The spectrum of evans syndrome in adults: new insight into the disease based on the analysis of 68 cases. Blood. (2009) 114:3167–72. doi: 10.1182/blood-2009-04-215368 19638626

[43] Zadro Y Lafaurie M Michel M Lapeyre-Mestre M Moulis G . Risk of hospitalization with infection in adults with primary AIHA treated with rituximab: a french nationwide study. Blood Adv. (2025) 9:231–8. doi: 10.1182/bloodadvances.2024013067 39189926 PMC11788127

[44] Huang H-E Lin K-M Lin J-C Lin Y-T He H-R Wang Y-W . Danazol in refractory autoimmune hemolytic anemia or immune thrombocytopenia: a case series report and literature review. Pharm (Basel). (2022) 15:1377. doi: 10.3390/ph15111377 36355549 PMC9692819

[45] Ni X Li D Yuan C Yu Y Wang H Wang L . Single-dose versus low-dose rituximab in corticosteroid-resistant or relapsed ITP: a multicenter, randomized, controlled study. Am J Hematol. (2022) 97:440–7. doi: 10.1002/ajh.26473 35049070

