# Supplementary material for: Clinical characteristics, treatment patterns and long-term outcomes of warm and mixed autoimmune hemolytic anemia: insights from a real-world study
Source: Front Immunol. 2026 Oct 12;17:1931581. doi: 10.3389/fimmu.2026.1931581 (PMC13711130; doi:10.3389/fimmu.2026.1931581)
Supplement: Supplementary file 1 [file Image1.pdf]

Supplementary Material

Supplementary Figure 1

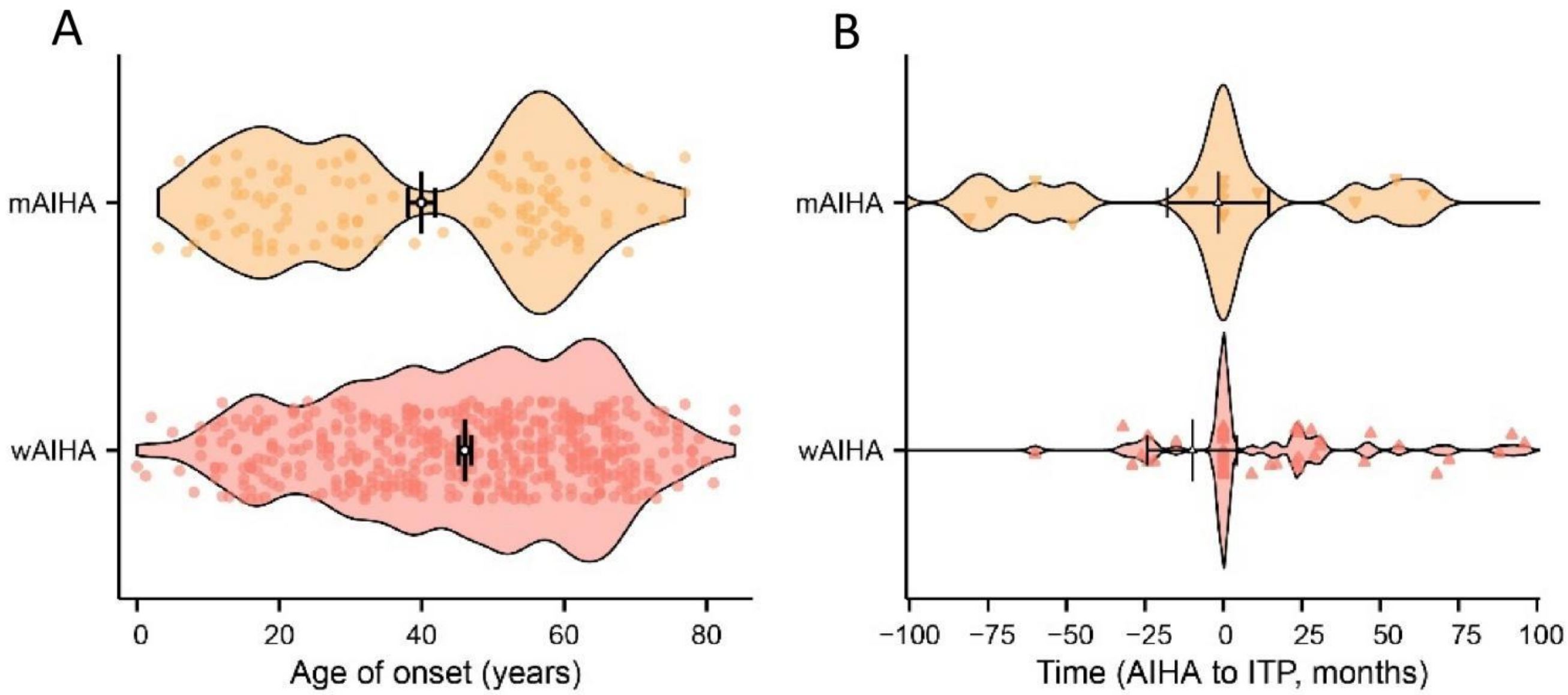

Supplementary Figure 1. Demographic profiles of the cohort. (A) Age distribution at disease onset stratified by clinical subtype (wAIHA vs. mAIHA). The mAIHA cohort exhibits a distinct bimodal age distribution with peaks at approximately 20 and 60 years. **(B)** Distribution of the temporal relationship between AIHA and ITP diagnosis in patients with Evans syndrome. **Abbreviations:** wAIHA, warm autoimmune hemolytic anemia; mAIHA, mixed autoimmune hemolytic anemia; ITP, immune thrombocytopenia.

Supplementary Figure 2

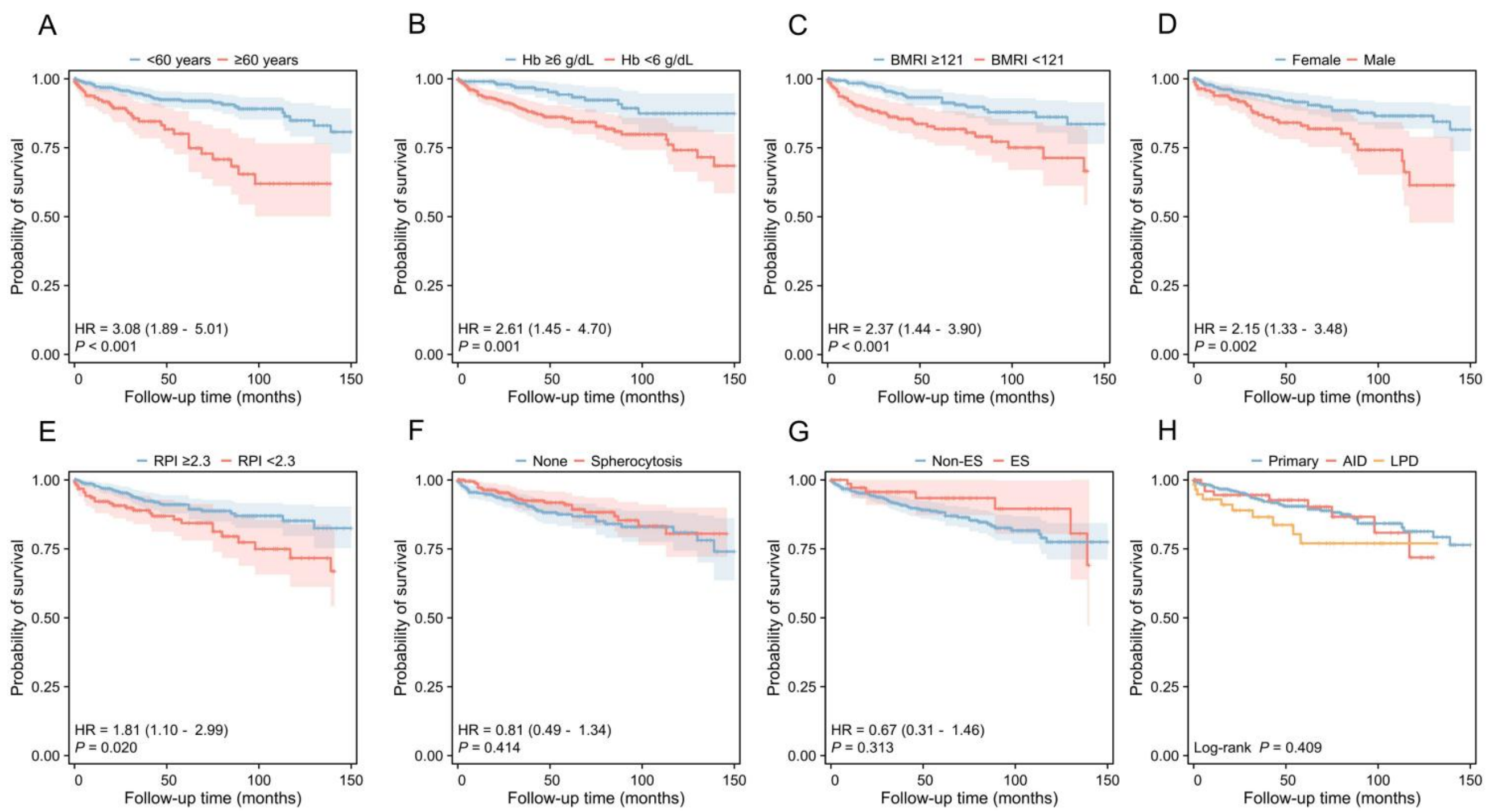

Supplementary Figure 2. Kaplan-Meier analysis of overall survival (OS) stratified by clinical characteristics. Comparisons include: **(A)** age (≥60 vs. <60 years); **(B)** hemoglobin at onset (<6 vs. ≥6 g/dL); **(C)** BMRI <121 vs. ≥121; **(D)** sex; **(E)** RPI <2.3 vs. ≥2.3; **(F)** spherocytosis; **(G)** Evans syndrome; and **(H)** etiology (primary AIHA vs. AIDs vs. LPDs). **Abbreviations**: BMRI, bone marrow responsiveness index; RPI, reticulocyte production index; AIDs, autoimmune/autoinflammatory diseases; LPDs, lymphoproliferative diseases.

Supplementary Figure 3

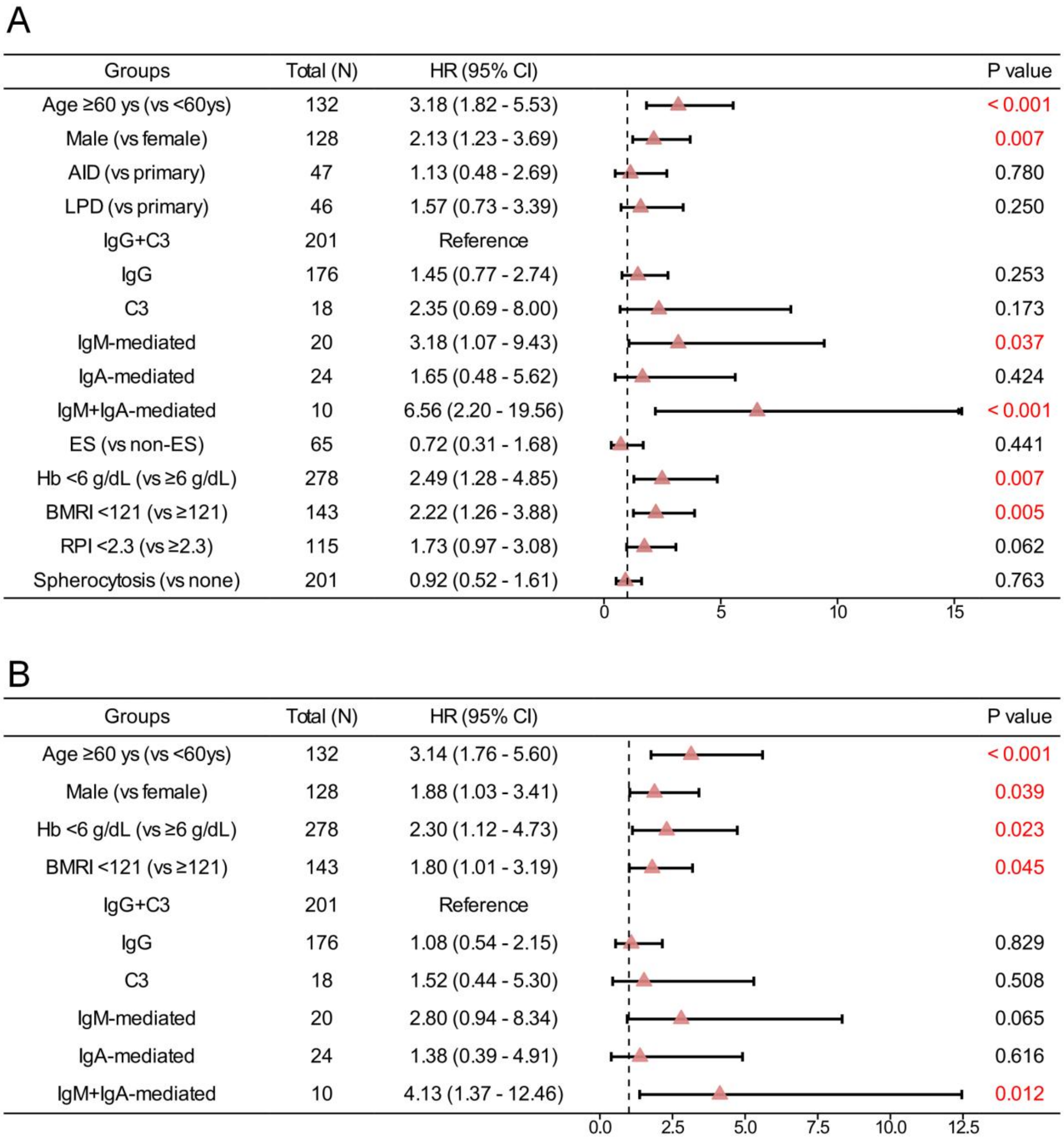

A

Groups	Total (N)	HR (95% CI)	P value
Age ≥60 ys (vs <60ys)	132	3.18 (1.82 - 5.53)	< 0.001
Male (vs female)	128	2.13 (1.23 - 3.69)	0.007
AID (vs primary)	47	1.13 (0.48 - 2.69)	0.780
LPD (vs primary)	46	1.57 (0.73 - 3.39)	0.250
IgG+C3	201	Reference	
IgG	176	1.45 (0.77 - 2.74)	0.253
C3	18	2.35 (0.69 - 8.00)	0.173
IgM-mediated	20	3.18 (1.07 - 9.43)	0.037
IgA-mediated	24	1.65 (0.48 - 5.62)	0.424
IgM+IgA-mediated	10	6.56 (2.20 - 19.56)	< 0.001
ES (vs non-ES)	65	0.72 (0.31 - 1.68)	0.441
Hb <6 g/dL (vs ≥6 g/dL)	278	2.49 (1.28 - 4.85)	0.007
BMRI <121 (vs ≥121)	143	2.22 (1.26 - 3.88)	0.005
RPI <2.3 (vs ≥2.3)	115	1.73 (0.97 - 3.08)	0.062
Spherocytosis (vs none)	201	0.92 (0.52 - 1.61)	0.763

B

Groups	Total (N)	HR (95% CI)	P value
Age ≥60 ys (vs <60ys)	132	3.14 (1.76 - 5.60)	< 0.001
Male (vs female)	128	1.88 (1.03 - 3.41)	0.039
Hb <6 g/dL (vs ≥6 g/dL)	278	2.30 (1.12 - 4.73)	0.023
BMRI <121 (vs ≥121)	143	1.80 (1.01 - 3.19)	0.045
IgG+C3	201	Reference	
IgG	176	1.08 (0.54 - 2.15)	0.829
C3	18	1.52 (0.44 - 5.30)	0.508
IgM-mediated	20	2.80 (0.94 - 8.34)	0.065
IgA-mediated	24	1.38 (0.39 - 4.91)	0.616
IgM+IgA-mediated	10	4.13 (1.37 - 12.46)	0.012

Supplementary Figure 3. Forest plots of Cox regression analyses for overall survival in patients with wAIHA. (A) Univariable Cox regression analysis of baseline clinical and laboratory variables. **(B)** Multivariable Cox regression analysis of baseline variables. Hazard ratios (HRs) with 95% confidence intervals (CIs) are presented, and statistically significant associations are highlighted in red. RPI was excluded from the multivariable analysis because of collinearity with BMRI. **Abbreviations:** Please refer to **Figure 4** for complete definitions of variables.

Supplementary Figure 4

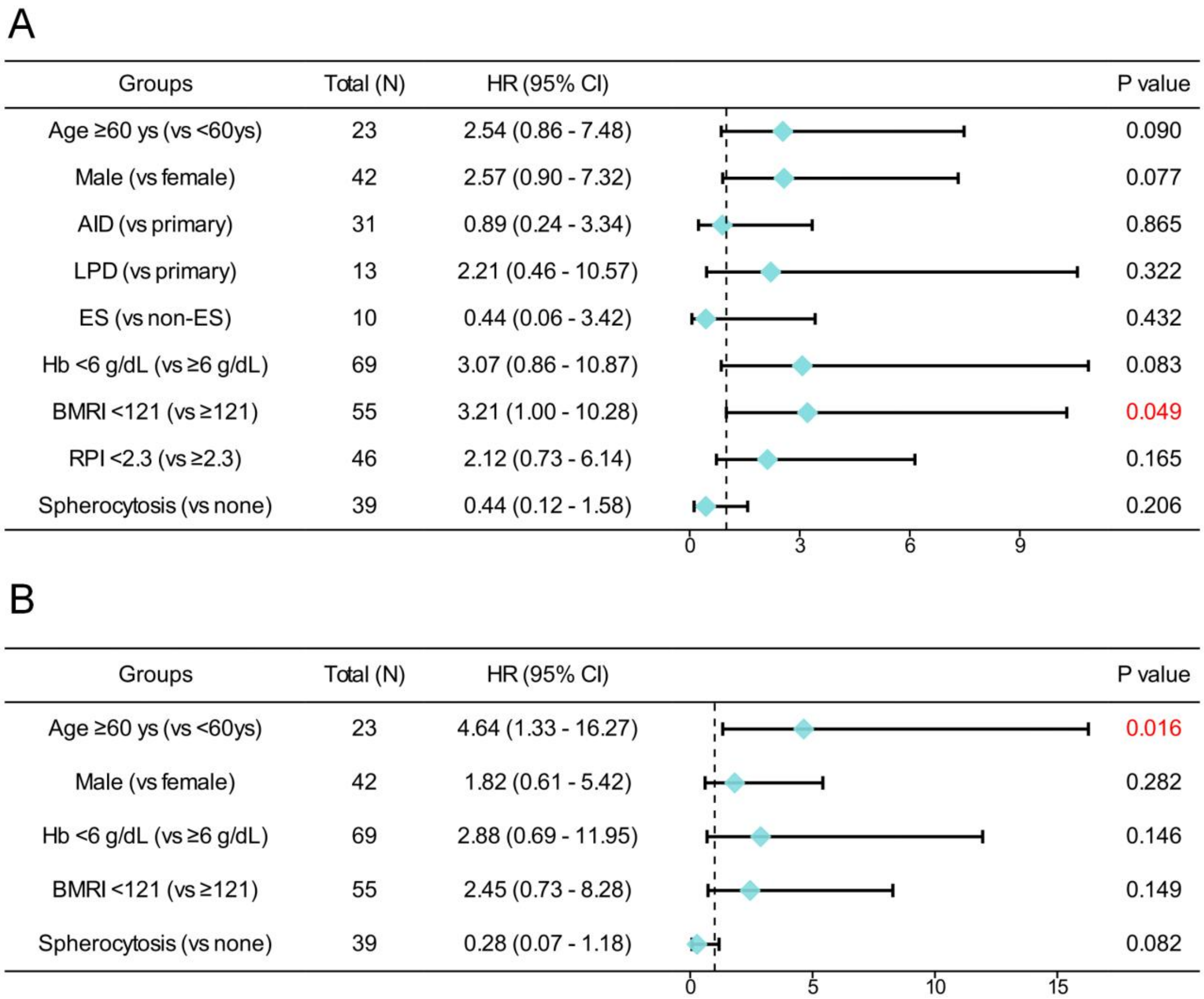

Supplementary Figure 4. Forest plots of Cox regression analyses for overall survival in patients with mAIHA. (A) Univariable Cox regression analysis of baseline clinical and laboratory variables. **(B)** Multivariable Cox regression analysis of baseline variables. Hazard ratios (HRs) with 95% confidence intervals (CIs) are presented, and statistically significant associations are highlighted in red. Serological subgroups were not included in the Cox regression analyses because of the small sample sizes within individual subgroups. RPI was excluded from the multivariable analysis because of collinearity with BMRI. **Abbreviations:** Please refer to **Figure 4** for complete definitions of variables.
