# Supplementary material for: Clinical characteristics, treatment patterns and long-term outcomes of warm and mixed autoimmune hemolytic anemia: insights from a real-world study
Source: Front Immunol. 2026 Oct 12;17:1931581. doi: 10.3389/fimmu.2026.1931581 (PMC13711130; doi:10.3389/fimmu.2026.1931581)
Supplement: Supplementary file 2 [file Table1.docx]

Supplementary Material

**Supplementary Table 1. Detailed distribution of underlying etiologies in the AIHA cohort.**

| **Etiology Category** | **Specific Disease / Subtype** | **n** |
| --- | --- | --- |
| Autoimmune diseases (AIDs) |  | 82 |
|  | Systemic lupus erythematosus | 45 |
|  | Sjögren's syndrome | 14 |
|  | Rheumatoid arthritis | 11 |
|  | Antiphospholipid syndrome | 9 |
|  | Autoimmune hepatitis | 6 |
|  | Other AIDs* | 6 |
| Lymphoproliferative diseases (LPDs) |  | 61 |
|  | B-cell LPDs | 41 |
|  | - CD5-CD10- small B-cell LPDs (unclassified) | 10 |
|  | - Marginal zone lymphoma* | 8 |
|  | - Lymphoplasmacytic lymphoma / Waldenström macroglobulinemia | 6 |
|  | - Chronic lymphocytic leukemia | 4 |
|  | - Diffuse large B-cell lymphoma | 4 |
|  | - CD5-CD10- large B-cell LPDs | 3 |
|  | - B-cell lymphoma (unspecified) | 2 |
|  | - Other B-cell LPDs* | 4 |
|  | T-cell LPDs | 14 |
|  | - T-cell large granular lymphocytic leukemia | 9 |
|  | - Angioimmunoblastic T-cell lymphoma | 2 |
|  | - T-cell LPDs (unclassified) | 3 |
|  | Other LPDs | 6 |
|  | - LPDs (unspecified) | 4 |
|  | - NK-cell LPD | 1 |
|  | - Castleman disease | 1 |
| Myeloid neoplasms |  | 5 |
|  | Myelodysplastic syndromes | 3 |
|  | Primary myelofibrosis | 2 |
| Other etiologies |  | 2 |
|  | Thymoma | 1 |
|  | Common variable immunodeficiency | 1 |
|  |  |  |
| Potential Immune Abnormalities* |  |  |
|  | Undifferentiated connective tissue disease | 54 |
|  | Autoimmune thyroiditis | 29 |
|  | Psoriasis | 2 |

Note: The sum of categorized etiologies slightly exceeds the total number of secondary AIHA patients (n=147) because three patients presented with concurrent etiologies across different categories (one with RA and T-LGLL, one with SLE and CD5-CD10- small B-LPD, and one with dermatomyositis and PMF). Similarly, several patients presented with multiple overlapping autoimmune disorders.

*Other AIDs include ankylosing spondylitis, systemic sclerosis, Behçet's disease, ulcerative colitis, dermatomyositis, and adult-onset Still's disease (n=1 for each).

*Marginal zone lymphoma (MZL) encompasses unclassified MZL (n=3), splenic marginal zone lymphoma (n=4), and MALT lymphoma (n=1).

*Other B-cell LPDs include follicular lymphoma, CD5-CD10- medium B-cell lymphoma, monoclonal gammopathy of undetermined significance, and multiple myeloma (n=1 for each).

*Patients with concurrent potential immune abnormalities were not classified as secondary AIHA based on these conditions alone. The numbers presented here reflect their total prevalence across both primary and secondary AIHA cohorts.
